# Myocardial injury and its correlation to mortality in hospitalized COVID-19 patients: A retrospective cohort study

**DOI:** 10.3389/fcvm.2022.1039655

**Published:** 2022-11-23

**Authors:** Muhannad J. Ababneh, Abdullah Al-Kasasbeh, Mohamad Jarrah, Lujain Malkawi, Omar Sanduka, Areje M. Smadi, Mahmoud M. Smadi

**Affiliations:** ^1^Division of Cardiology, Department of Internal Medicine, Faculty of Medicine, Jordan University of Science and Technology, Irbid, Jordan; ^2^Department of Internal Medicine, Faculty of Medicine, Jordan University of Science and Technology, Irbid, Jordan; ^3^Faculty of Medicine, Jordan University of Science and Technology, Irbid, Jordan; ^4^Department of Mathematics and Statistics, Jordan University of Science and Technology, Irbid, Jordan

**Keywords:** SARS-CoV-2, COVID-19, troponin, myocardial infarction, acute kidney injury, risk stratification

## Abstract

**Purpose:**

This retrospective observational study was conducted to assess the clinical characteristics and outcomes of hospitalized COVID-19 patients with positive cardiac enzymes in the King Abdullah University Hospital (KAUH) in Irbid, Jordan.

**Patients and methods:**

A total of 676 patients admitted to KAUH for moderate-to-severe COVID-19 were included in the study. Clinical and mortality data were collected from patients’ electronic medical records.

**Results:**

A significant association was found between myocardial injury and In-hospital mortality. Seven comorbidities were identified as risk factors for myocardial injury: Hypertension, diabetes mellitus (DM), previous cerebrovascular accident (CVA), ischemic heart disease (IHD), heart failure, chronic kidney disease (CKD), and cardiac arrhythmias. The need for intensive care unit (ICU) for invasive ventilation was also associated with myocardial injury. Acute kidney injury (AKI) during hospitalization had a significantly higher incidence of myocardial injury and mortality. Acute myocardial infarction (MI) and acute peripheral vascular disease (PVD) were also associated with higher mortality.

**Conclusion:**

Myocardial injury is an important predictor of mortality in patients with moderate-to-severe COVID-19 disease. Patients with a history of hypertension, diabetes mellitus, any vascular diseases, cardiac arrhythmias or heart failure are considered high-risk for adverse outcome. Additionally, COVID-19 patients with myocardial injury and acute kidney injury were recognized with the highest mortality rate.

## Introduction

The COVID-19 pandemic remains to be a global health crisis. Attempts from the scientific community to better understand this disease continue to reveal new characteristics of the novel coronavirus (SARS-CoV-2), ranging from diagnostic challenges to disease mechanisms. Initially thought of as a solely respiratory disease, manifestations of COVID-19 in different organ systems are constantly being reported. While several healthcare professionals and institutions have described vascular thrombotic complications (1), other studies reporting gastrointestinal (2), neurological (3), and cardiac (4) events have emerged. Despite the growing body of literature on the cardiovascular manifestations of moderate-to-severe COVID-19 disease, many questions remain unanswered. More research is needed to better understand the risk, mechanisms, and outcomes of cardiac disease associated with COVID-19 infections.

In the current literature, there has been increasing evidence suggesting cardiovascular involvement in COVID-19 disease. A recent meta-analysis of 35 studies has estimated the frequency of newly developed acute cardiac injury in COVID-19 patients to be more than 25%, in addition to other manifestations such as heart failure and arrhythmias (5). Similar evidence has led other studies to investigate elevation in cardiac biomarkers, such as troponin I, as a potential prognostic factor. A meta-analysis of 10 studies found that 51% of patients with poor outcomes had elevated serum troponin I levels, suggesting that troponin I, and possibly other cardiac biomarkers, could be utilized as a valuable predictor of severe COVID-19 disease (6). Poor outcomes were defined by need for intensive care unit (ICU) admission, oxygen saturation below 90%, invasive mechanical ventilation and in-hospital mortality (6). Another study of 100 patients who had echocardiographic investigations found that 39% of patients had right ventricular dilation and dysfunction and that elevated troponin I levels were associated with worse right ventricular function (7). The mechanisms of myocardial injury in COVID-19 infections remain unclear. There have been a few case reports of COVID-19 infections complicated by myocarditis, though there is no evidence ruling out other pathologies such as supply/demand mismatch and systemic inflammation (8, 9). It is important to note that myocardial injury has been observed in cases of acute respiratory distress syndrome (ARDS) unrelated to the novel coronavirus. A prospective cohort study in 2017 revealed that high-sensitivity troponin I (hsTroponin I) was detectable in 94% of patients with ARDS (10). This suggests that direct cardiotoxicity may not be necessary for myocardial injury in COVID-19 patients, since ARDS in general can cause an elevation in serum hsTroponin I. Despite the increasing evidence of myocardial injury in COVID-19 infections, current understanding of the prognostic value of such findings is quite poor. This calls for further research to assess both the outcomes and initial clinical characteristics of those patients. Additionally, none of the studies assessing cardiac biomarkers in COVID-19 patients was conducted in the MENA region (Middle East and North Africa), which indicates that studies in the region are needed to improve our understanding of this disease and its impacts on this population, and to make similar results obtained from other researcher generalisable.

## Materials and methods

In this work, the retrospective cohort study approach was adopted for analysis of COVID-19 patients hospitalized in King Abdullah University Hospital (KAUH). King Abdullah University Hospital (affiliated with Jordan University of Science and Technology), is a tertiary referral center that serves patients from North of Jordan, the majority of them are Arabs. The indication for admission were the existing of severe hypoxia defined as O_2_ saturation below 90% or patients with other comorbidities and clinical instability. The targeted group were investigated for elevated troponin I levels and/or electrocardiogram (ECG) abnormalities during the period between 1 September and 31 December 2020. Data were collected from the patients’ electronic medical records. The parameters used in the study included demographic information, hospital admission duration, full medical history including presenting illness, past medical and surgical history, physical exam findings, vital signs, ECG findings, serum troponin I (normal range 0.0–0.02 ng/ml) and other biomarker levels [c reactive protein (CRP), and creatine kinase MB (CK-MB)], intensive care unit (ICU) admission, use of mechanical ventilation, occurrence of acute complications during hospitalization (cardiac infarction, cerebral or peripheral vascular complications, and acute kidney injury), and in-hospital mortality.

Descriptive statistics were used to summarize and determine the sample patients’ characteristics and distribution of patients’ data. Among descriptive statistics, we determined the range, mean, standard deviation (S.D), and median for the continuous variables and counts, percentages, and cross tabulation for the categorical variables. Also, we presented visual examination of data using clustered bar charts and error bar charts. Inferential statistics were also used to assess associations between categorical variables and group comparisons between quantitative variables. Associations between two or more qualitative data variables were assessed using Fishers exact test and Cramer’s V test. Quantitative data between more independent groups were analyzed using two independent samples *t*-test and One-way ANOVA procedure. Multivariate binary logistic regression was conducted to assess the influence of different qualitative and quantitative risk variables associated with different outcomes. All *P*-values presented were two-tailed, and *P*-values < 0.05 were considered as statistically significant. All statistical analyses were performed using statistical package SPSS 21.0 (SPSS Inc., Chicago, IL).

## Results and discussion

### Patients’ characteristics

**FIGURE 1:**
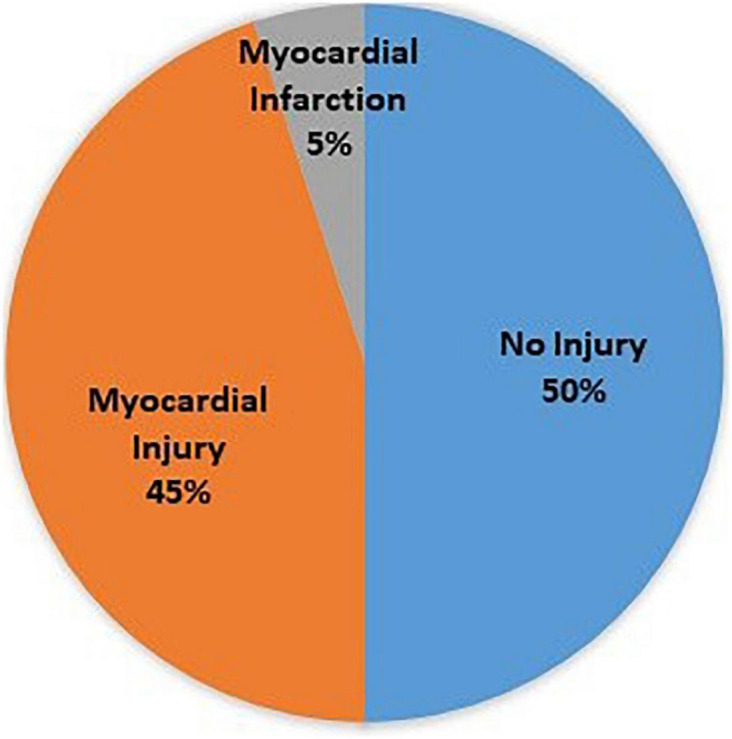
Pie chart of patient groups (no injury, injury, and infarction).

A total of 676 patients were included in the study, 56.8% of which were males. Their ages ranged between 16 and 96 years, with a mean of 64.50 ± 13.99 years. Analysis of the data showed that 38 patients are smokers (5.7%), including 6 female smokers (2.1%) out of 292 female patients and 32 male smokers (8.4%) out of 384 male patients. The recorded comorbidity rates of hypertension (HTN), diabetes mellitus (DM), pre-diabetes mellitus (pre-DM), pulmonary disease, hypercholesterolemia, ischemic heart disease (IHD), previous cerebrovascular accident (CVA), peripheral vascular disease (PVD), and chronic kidney disease (CKD) are shown in Table 1. Patients with positive cardiac enzymes were 50% of all included patients; 5% were in the myocardial infarction group including STEMI and NSTEMI (infarction group), and 45% were considered as myocardial injury group (injury group), the remaining 50% without elevation in cardiac enzymes were considered as no injury group. The Pie chart in Figure 1 demonstrates the distribution in the three study groups.

**TABLE 1:** Demographic characteristics of patients.

Variable		Count (%)
Gender	Male	384 (56.8%)
	Female	292 (43.2%)
Smoking status	Yes	38 (5.7%)
	No	631 (94.3%)
Hypertension	Yes	433 (64.1%)
	No	236 (34.9%)
Diabetes mellitus	Yes	350 (51.8%)
	No	319 (47.2%)
Pre-diabetes mellitus	Yes	11 (1.6%)
	No	658 (98.4)
Hypercholesterolemia	Yes	31 (4.6%)
	No	638 (95.4%)
Pulmonary disease	Yes	46 (6.9%)
	No	623 (93.1%)
Previous cerebrovascular accident	Yes	51 (7.6%)
	No	618 (92.4%)
Ischemic heart disease	Yes	139 (20.6%)
	No	530 (78.4%)
Peripheral vascular disease	Yes	21 (3.1%)
	No	648 (96.9%)
Heart failure	Yes	76 (11.4%)
	No	593 (88.6%)
Chronic kidney disease	Yes	86 (12.9%)
	No	583 (87.1%)

### Characteristics of patients with positive cardiac enzymes

The patients were categorized into three groups according to their serial cardiac enzymes levels and clinical history notes. Patients with elevation in their cardiac enzymes without evidence of Myocardial infarction (MI) were categorized into the “injury” group. Patients with elevation in their cardiac enzymes in addition to clinical evidence of MI were classified as the “infarction” group. Clinical evidence of MI is defined as the presence of typical chest pain with ST elevation in ECG, ischemic ECG changes or changes in myocardial function (echocardiography). Patients who did not have elevation in their cardiac enzymes or evidence of infarction were set as the “no injury” group. A univariate analysis was conducted to assess the effect of different predictors on myocardial injury using Fisher exact test; the results are shown in Table 2. The following predictors were found to be statistically significant in predicting myocardial injury: Hypertension (*p*-value < 0.001), diabetes mellitus (*p*-value = < 0.001), previous CVA (*p*-value = 0.008), IHD (*p*-value < 0.001), HF (*p*-value < 0.001), CKD (*p*-value < 0.001), and pre-existing arrhythmia (*p*-value = 0.005).

**TABLE 2:** Association between patient groups (no injury, injury, and infarction) and patients’ characteristics.

Variable		Patient group			*P*-value
		No injury	Injury	Infarction	
Gender	Male	182	185	17	0.11
	Female	160	117	15	
Hypertension	Yes	192	217	24	<0.001
	No	144	84	8	
Diabetes mellitus	Yes	149	179	22	<0.001
	No	187	122	10	
Pre-diabetes mellitus	Yes	8	3	0	0.295
	No	328	298	32	
Smoking status	Yes	16	21	1	0.394
	No	320	280	31	
Hypercholesterolemia	Yes	14	14	3	0.408
	No	322	287	29	
Pulmonary disease	Yes	24	22	0	0.288
	No	312	279	32	
Previous cerebrovascular accident	Yes	15	33	3	0.008
	No	321	268	29	
Ischemic heart disease	Yes	46	81	12	<0.001
	No	290	220	20	
Peripheral vascular disease	Yes	7	11	3	0.061
	No	329	290	29	
Heart failure	Yes	12	56	8	<0.001
	No	324	245	24	
Chronic kidney disease	Yes	12	71	3	<0.001
	No	324	230	29	
Pre-existing arrhythmia	Yes	11	20	5	0.005
	No	331	282	27	

Multivariate binary logistic regression analysis was performed to assess the significance of a combination of different risk factors on myocardial injury (1: injury, 0: no injury). The candidate risk factors considered in the analysis were: Age, Hypertension (HTN), diabetes mellitus (DM), Smoking status, Hypercholesterolemia, pulmonary disease, previous cerebrovascular accident (CVA), ischemic heart disease (IHD), peripheral vascular disease (PVD), heart failure (HF), chronic kidney injury (CKD), and Pre-existing arrhythmia. A significant logistic regression equation was found using 14 predictors with an Nagelkerke *R*^2^ = 39.3%. The significant predictors for myocardial injury were gender, age, HF, DM, and CKD.

A univariate analysis was conducted to assess the effect of different predictors (patient admission for >24 h, ICU admission, and invasive ventilation) on the patient groups (injury, infarction, and no injury) using Cramer’s V test. The results are shown in Table 3. The following variables were found to be statistically significant predictors of myocardial injury: ICU admission and invasive ventilation with *p*-values < 0.001. Using one-WAY ANOVA procedure, significant differences were found for O_2_ saturation on admission, while no significant differences for the remaining vital signs on admission (respiratory rate, temperature, systolic blood pressure, and diastolic blood pressure), as depicted in Table 3.

**TABLE 3:** Association and group comparisons between patient groups (injury, infarction, and no injury) and ICU admission, hospital admission for >24 h, invasive ventilation, and vital signs on admission.

Attribute		*n* (%)	Patient groups			*P*-value
			Injury	Infarction	No injury	
Patient admitted for >24 h	Yes	646 (97.14%)	290	32	324	0.401
	No	19 (2.86%)	7	0	12	
ICU admission	Yes	176 (26.4%)	108	17	51	<0.001
	No	485 (73.6%)	185	15	285	
Invasive ventilation	Yes	127 (19.33%)	79	10	38	<0.001
	No	530 (80.67%)	215	20	295	
Respiratory rate on admission		***N***	266	27	302	0.072
		Mean value	21.97	21.22	21.46	
		S.D	2.79	2.819	2.818	
Temperature on admission		***N***	290	30	330	0.143
		Mean value	37.16	37.22	37.28	
		S.D	0.748	0.646	0.99	
Systolic blood pressure on admission		***N***	297	31	31	0.108
		Mean value	130.86	129.14	129.16	
		S.D	22.316	19.295	19.413	
Diastolic blood pressure on admission		***N***	297	31	330	0.368
		Mean value	74.94	75.58	76.1	
		S.D	11.92	9.605	8.563	
O_2_ saturation on admission		***N***	258	30	309	<0.001
		Mean value	85.2	85.30	89.4	
		S.D	12.099	11.052	7.051	

*N*, number of patients; S.D, standard deviation.

**FIGURE 2:**
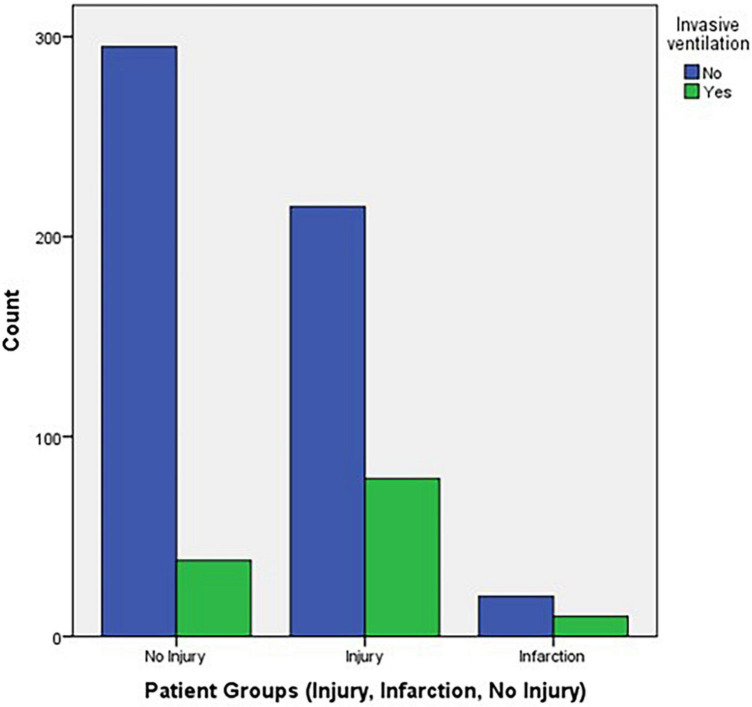
Stacked bar chart of invasive ventilation vs. patient groups.

In the stacked bar chart in Figure 2 we can demonstrate a significant correlation for the need of invasive mechanical ventilation in patients with myocardial involvement (injury of infarction).

**FIGURE 3:**
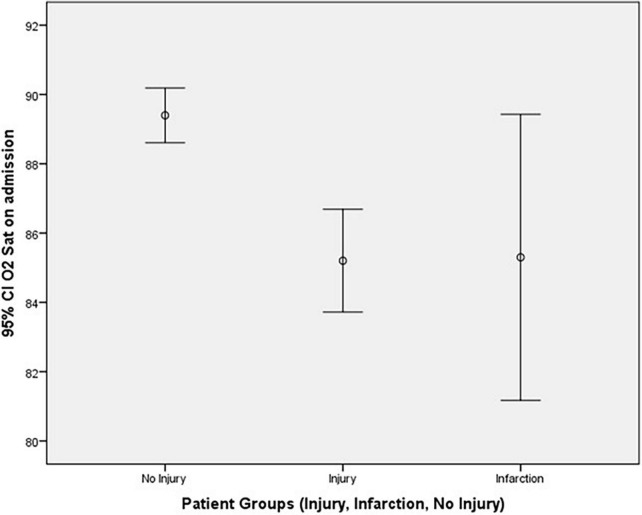
Error bar plot of O_2_ saturation on admission against patient groups.

Error bar plot of O_2_ saturation on admission in the different patient groups is also shown in Figure 3.

### Mortality and patient groups (injury, no injury, STEMI, and NSTEMI)

The “infarction” patient group was subdivided into two further groups according to their ECG findings: ST segment elevation MI (STEMI) and non-ST segment elevation MI (NSTEMI). A contingency table of mortality vs. patient groups (injury, no injury, STEMI, and NSTEMI) is shown in Table 4. A significant association between mortality and patient groups was detected with *p*-value < 0.001 using Cramer’s V test.

**TABLE 4:** Contingency table of mortality and patient groups (injury, no injury, STEMI, and NSTEMI).

	Counts and %	Patient groups				Total
		No injury	Injury	STEMI	NSTEMI	
Mortality	Count	58	142	5	13	218
	% within patient group	17.0%	47.0%	83.3%	50.0%	32.2%
	% of total patients	8.6%	21.0%	0.7%	1.9%	32.2%
	% of mortality	26.6%	65%	2.3%	6%	100%

### Mortality with intensive care unit admission and invasive ventilation

To study the association between mortality and patients’ clinical findings, a univariate analysis was conducted to assess the effect of different predictors (patient admission for >24 h, ICU admission, and invasive ventilation) on the mortality using Fishers exact test. The results are shown in Table 5. The following predictors were found to be statistically significant in predicting mortality: Patient admission for >24 h (*p*-value = 0.025), ICU admission, and invasive ventilation with *p*-values < 0.001.

**TABLE 5:** Association and group comparisons of mortality with hospital admission for >24 h, ICU admission, and invasive ventilation.

Variable		*n* (%)	Mortality		*P*-value
			No	Yes	
Patient admitted for >24 h	Yes	646 (97.14%)	430	216	0.025
	No	19 (2.86%)	17	2	
	Total	665			
ICU admission	Yes	176 (26.63%)	19	157	<0.001
	No	485 (73.37%)	428	57	
	Total	661			
Invasive ventilation	Yes	127 (19.33%)	5	122	<0.001
	No	530 (80.67%)	440	90	
	Total	657			

Multivariate binary logistic regression analysis shows the significance of the risk factors patient admission for >24 h, ICU admission, and invasive ventilation on mortality and yields Nagelkerke measure of *R*^2^ = 67.9%.

### Mortality and myocardial injury in patients with acute complications

The incidence of acute MI, acute kidney injury (AKI), acute peripheral vascular disease (PVD), and acute CVA was recorded. Table 6 demonstrates the incidence of acute complications and mortality during patients’ hospital stay:

**TABLE 6:** Incidence of acute complications and mortality.

Mortality	Yes	218 (32.2%)
	No	458 (67.8%)
Acute MI	Yes	20 (3%)
	No	641 (97%)
Acute CVA	Ischemic CVA	5 (0.8%)
	Unspecified CVA	3 (0.5%)
	No CVA	654 (98.8%)
Acute peripheral vascular disease (PVD)	Yes	11 (1.7%)
	No	652 (98.3%)
Acute kidney injury (AKI)^a^	Yes	102 (15.4%)
	No	560 (84.6%)

^a^AKI as defined by KDIGO—as an increase in baseline serum creatinine of either 1.5 times over 7 days or 26.5 mmol/L over 48 h, or documented oliguria (urine output of <0.5 mL/kg/h).

Statistical analyses were performed to investigate the association between the incidence of acute complications during hospitalization with myocardial injury (described as the three patient groups) (Table 7) and mortality (Table 8). Using Cramer’s V test, the incidence of acute kidney injury (AKI) was significantly associated with myocardial injury (*p*-value = <0.001).

**TABLE 7:** Acute complications and patient groups (no injury, injury, and infarction).

Acute complication	Incidence	Patient group			*P*-value
		No injury	Injury	Infarction	
Acute kidney injury (AKI)^a^	Yes	20	71	11	<0.001
	No	315	224	21	
Acute CVA	Ischemic CVA	1	4	0	0.52
	Unspecified CVA	1	2	0	
	No	332	290	32	
Acute PVD	Yes	5	6	0	0.656
	No	330	290	32	

^a^AKI as defined by KDIGO—as an increase in baseline serum creatinine of either 1.5 times over 7 days or 26.5 mmol/L over 48 h, or documented oliguria (urine output of <0.5 mL/kg/h).

**TABLE 8:** Acute complications and mortality.

Complication		Mortality		*P*-value
		Yes	No	
Acute MI	Yes	16	4	<0.001
	No	205	446	
Acute Kidney Injury (AKI)^a^	Yes	79	25	<0.001
	No	143	425	
Acute CVA	Ischemic	3	2	0.194
	Unspecified	2	1	
	No	215	448	
Acute PVD	Yes	7	4	0.034
	No	214	448	

^a^AKI as defined by KDIGO—as an increase in baseline serum creatinine of either 1.5 times over 7 days or 26.5 mmol/L over 48 h, or documented oliguria (urine output of <0.5 mL/kg/h).

Using Fisher’s exact test, a significant association was found between mortality and the incidence of acute MI, AKI, and acute PVD. No significant association was found between mortality and acute CVA using Cramer’s V test. The results are displayed in Table 8.

## Conclusion

The study demonstrates that myocardial injury, represented by elevation in serum troponin I, is an important predictor of mortality in patients with moderate-to-severe COVID-19 disease. Specifically, patients who are expected to suffer myocardial injury according to the findings are those with a medical history of hypertension, DM, CVA, IHD, heart failure, CKD, or cardiac arrhythmias. An early testing for elevated cardiac biomarkers (troponin I) can be used as a predictor for adverse outcome and complications and help in patients triage and need for admission.

The study also suggests that patients who require ICU admission, invasive ventilation, or both should have their serum troponin I levels monitored regularly during their hospital stay, as these readings can be a significant predictor of mortality.

The incidence of AKI during hospitalization was identified as a poor prognostic factor, as it was highly associated with myocardial injury and a higher In-hospital mortality. Other complications that were associated with higher In-hospital mortality were acute MI and acute PVD.

According to the findings of this study and considering the reports of cardiac complications of COVID-19 in the literature, a thorough investigation into the mechanisms of myocardial injury in COVID-19 disease would be recommended. We would also suggest further research of the incidence, pathophysiology, and potential predictors of AKI in COVID-19 patients.

### Limitations

The authors believe that the prevalence of smokers in this study (5.7%) was underreported; a 2014 national survey by the World Health Organization found the prevalence of smokers in the Jordanian population to be around 32.3% (11). We hypothesize that this underestimation was due to underreporting, or incomplete documentation in the electronic records. The authors also believe that the prevalence of hypercholesterolemia in the study (4.6%) was below the actual figure due to lack of primary screening in the local community.

## Data availability statement

The raw data supporting the conclusions of this article will be made available by the authors, without undue reservation.

## Ethics statement

The collection and use of patients’ data was approved by the Institutional Review Board (IRB) of Jordan University of Science and Technology (JUST) and King Abdullah University Hospital (KAUH). Written informed consent for participation was not required for this study in accordance with the national legislation and the institutional requirements.

## Author contributions

MA: primary investigator, IRB proposal, supervising data collection and analysis, and manuscript writing and editing. AA-K and MJ: supervising data collection and manuscript preparation. LM: IRB proposal, supervising data collection and manuscript preparation. OS: data collection, and manuscript writing and editing. AS: statistical analysis and manuscript editing. MS: supervising data collection, statistical analysis, and manuscript preparation. All authors contributed to the article and approved the submitted version.

## Abbreviations

KAUH: King Abdullah University Hospital
HTN: hypertension
DM: diabetes mellitus
MI: myocardial infarction
CKD: chronic kidney disease
IHD: ischemic heart disease
PVD: peripheral vascular disease
CVA: cerebrovascular accident
AKI: acute kidney injury
ICU: intensive care unit
STEMI: ST elevation myocardial infarction
NSTEMI: non-ST elevation myocardial infarction.
